# Nanotechnology‐enhanced edible coating application on climacteric fruits

**DOI:** 10.1002/fsn3.2557

**Published:** 2022-04-20

**Authors:** Temitayo Odetayo, Samson Tesfay, Nomali Ziphorah Ngobese

**Affiliations:** ^1^ Department of Botany and Plant Biotechnology Faculty of Science University of Johannesburg Johannesburg South Africa; ^2^ Department of Horticultural Science Faculty of Science University of KwaZulu‐Natal South Africa Pietermaritzburg South Africa

**Keywords:** nanoparticles, plant extracts, postharvest, shelf life

## Abstract

Climacteric fruits continue to ripen after harvest and produce ethylene, coupled with an increase in respiration rate, which contributes to more rapid perishability. Inhibition of ethylene biosynthesis has been shown to be an efficient way to delay the onset of ripening and lengthen shelf life. The use of edible materials as coatings presents an efficient approach in preserving the quality of fruits. Edible coatings have many benefits, such as affordability, ease of application, and use of natural ingredients. Nanotechnology provides interesting approaches to the management of fruit shelf life after harvest. Nanotechnology has the capacity of producing new materials by minimizing the size of components to a nanometric level. These kinds of nanomaterials possess distinct and improved properties for delaying fruit ripening and decay. The main goal of adding nanoparticles to edible coatings is to enhance the biopolymer's mechanical and water vapor barrier properties. Nanoparticles also contain biopolymer‐like features and are thought to have superior antibacterial, antifungal, and antiviral properties than edible coatings. This review is aimed at summarizing recent findings on the application of edible coatings in the form of nanoparticles, and their effect on quality parameters and shelf life extension of climacteric fruits. Peer‐reviewed articles were obtained by using Scopus and science direct. The current materials widely used for coating climacteric fruits are zinc, silver and chitosan nanoparticles. Zinc nanoparticles have been shown to be more effective in delaying ripening significantly by reducing weight and moisture loss and ensuring retention of fruit firmness. Further research is needed to understand their effect on other physicochemical properties of fruits.

## Graphical abstract

The article reports on advances in the use of edible coatings in the fresh fruits and vegetables industry and the possibilities offered by nanotechnology in developing nanoparticle‐enhanced formulations. The manuscript specifically describes the basal components of such coatings, highlighting the strengths and shortfalls of popular plant‐based non‐enhanced coatings.

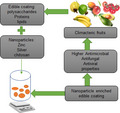

## 1 INTRODUCTION

Technologies such as ozone treatment (Alencar et al., 2013; Ali et al., 2014), modified atmosphere (Lalel et al., 2005; Lanka et al., 2011; Prange et al., 2013), 1‐Methylcyclopropene (De Martino et al., 2007a; De Martino et al., 2007b; Mir et al., 2004; Razzaq et al., 2016), controlled atmosphere storage (Hailu & Worku, 2017), and low‐temperature storage (Kudachikar et al., 2011) have been used in controlling ripening in climacteric fruits. For several years up to date, low‐temperature storage is used commercially in the supply and marketing chain from precooling at the farm to using refrigerated trucks set at a certain temperature for transportation to cold rooms at the fresh produce market (Zhang et al., 2016). Although low‐temperature storage has been successful in controlling ripening in climacteric fruits, storing fruit at suboptimal temperatures can cause chilling injury leading to internal browning and emergence of black spots (Sivankalyani et al., 2015). Other postharvest technologies have also been successful, but some have certain disadvantages, such as a reduction in the volatile esters in fruit and a negative impact on the nutritional properties of fruit (Skog & Chu, 2001). Furthermore, the application of controlled and modified atmosphere is expensive, and it involves specialized labor, thereby limiting its practical application. Use of 1‐Methylcyclopropene on climacteric fruit might induce chilling injury and require additional time to ripen adequately, limiting its economic applicability (Harris et al., 2000; Mir et al., 2004; De Martino et al., 2007a; De Martino et al., 2007b).

Edible coating is another type of technology becoming popular for controlling the ripening of climacteric fruits because coatings are simple to prepare, widely available, relatively low cost and mostly do not need the use of sophisticated atmosphere and temperature control technologies (Jodhani & Nataraj, 2021; Suhaget al., 2020). Edible coatings are thin layers of edible material added on the surface of climacteric fruits to enhance the appearance, maintain quality, prevent microbial growth, reduce respiration, slow maturation, minimize water loss, and extend shelf life (Murmu & Mishra, 2018). Besides, the use of edible coatings in fresh produce has been approved by the US Food and Drug Administration and is considered to meet the GRAS (generally regarded as safe) requirements (Dhall, 2013; Martín‐Belloso et al., 2009). To comply with export protocols, all the processes involved in the application of edible coatings must follow the high hygiene requirements and the quantity used must not exceed the amount necessary to achieve the desired physical and nutritional impact on the fruit (Nussinovitch & Nussinovitch, 2003). However, some edible coatings can pose disadvantages when used on fruits: for instance, edible coating formulated from polysaccharides and proteins exhibits weak water vapor barrier properties while lipids and waxes exhibit poor mechanical characteristics; thus, new alternative methods have been developed to solve these drawbacks.

**FIGURE 1:**
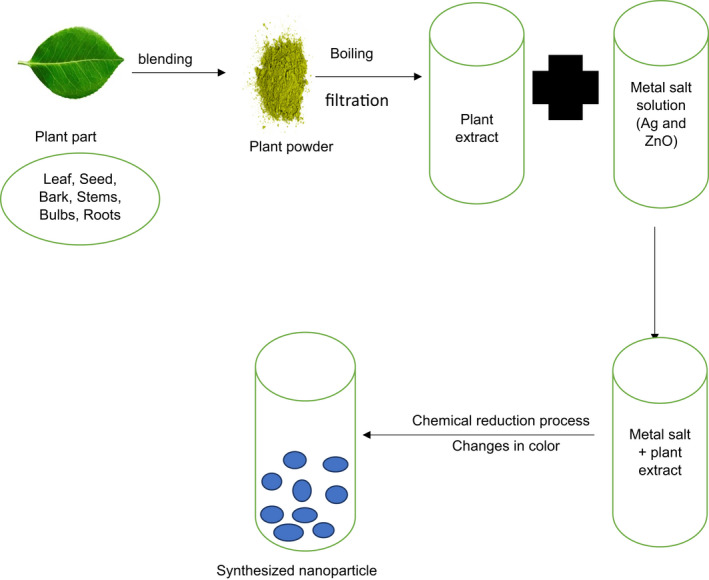
Schematic representation of nanoparticle synthesis using green method (Esa & Sapawe, 2020)

Nanotechnology is a novel approach for creating new edible coating components for the postharvest management of fresh produce by reducing the size of the edible coating particles to a nanometric scale ranging from 1 to 100 nanometers (Parisi et al., 2015). The application of nanotechnology has proven to be one of the best strategies for extending the shelf life of fresh fruits and nanoparticles used for edible coatings also possesses unique properties such as antibacterial and antifungal qualities (Bajpai et al., 2018; Lloret et al., 2012; Singh et al., 2020). Recently, there have been studies on the effects of the addition of nanoparticles to edible coatings and these studies have revealed that the addition of nanoparticles to edible coatings has opened new opportunities not only for enhancing higher antimicrobial, antifungal, and antiviral properties, but also to improve the cost‐effectiveness of edible coatings (Sorrentino et al., 2007). The reviews by Nor and Ding (2020), Dhaka and Upadhyah (2018), and Ncama et al.  (2018) have compiled all possible coatings that are functional for tropical fruits and horticultural produce, highlighting safe concentrations, and the use of commonly consumed plant extracts that have potential to be used on fresh produce. The above reviews cover the use of polysaccharides, proteins, and lipids as edible coatings on popular fruits. The reviews also show that edible coatings are a promising method for extending the postharvest shelf life of tropical fruits. The current review talks specifically to the use of nanoparticle materials in addition to edible coating on climacteric fruits. Recent review studies have reported the successful application of nanotechnology in the food packaging industry, agricultural sector, agri‐food sectors as well as in the postharvest disease management of fruits and vegetables (Al‐Tayyar et al., 2020; Chawla et al., 2021; Lang et al., 2021; Mahela et al., 2020; Ruffo Roberto et al., 2019; Wahab et al., 2021). As a result of all these studies, the addition of various kinds of nanoparticles to the different edible coatings to maintain the quality of food appears to be a viable consideration. However, there is still limited information about the effectiveness of nanoparticle edible coating material in regard to their application to climacteric fruits. Therefore, this review aims to highlight the most relevant and recent information on the use of edible coatings enriched with chitosan, silver, and zinc nanoparticles in extending postharvest storage life and the overall preservation of the quality of popular climacteric fruit (Figure 1).

## 2 PROBLEMS FACED BY CLIMACTERIC FRUITS

Based on their respiration behavior and ethylene production, fruits such as banana, mango, guava, apricot, pear, papaya, apple, avocado, tomato, and plantain are referred to as climacteric fruits (Atkinson et al., 2011). Climacteric fruits are usually harvested at physiological maturity and remain firm without major changes in peel color, texture, or composition before the beginning of maturation (Mendoza & Aguilera, 2006). After harvesting, the fruits undergo progressive deterioration, resulting in a relatively short postharvest life, increased respiratory rate, autocatalytic ethylene development, increase in susceptibility to various pathogenic infections, and sensory changes such as color, taste, and texture change such as softening (Palapol et al., 2009; Paul et al., 2012).

In postharvest fruit management, the perishability of fruit after harvest is a major challenge faced by the industry influencing produce marketability, especially for international trade (Singh et al., 2014). Globally, a massive quantity of fruits are wasted before the commodity reaches consumers, with about 50% of those food losses being valuable fruits. The U.S.D.A. Economic Research Service reports that over 34.6% of the loss is directly related to unwanted climacteric maturation, resulting in subsequent spoilage, degradation, mechanical injuries, and physiological disorders in produce (Barth et al., 2009; Kader, 2004). The perishability of the fruits is mainly attributable to adverse physicochemical changes, such as loss of weight due to respiration, softening of the flesh, deterioration of quality due to microbial attack, and changes in the content of sugar and acid. Another big threat to the global fruit supply chain is contamination by fungal pathogens. Postharvest fruit loss due to phytopathogens like fungi now accounts for more than half of all agricultural fruit production (Zhang et al., 2017). The most important factors causing postharvest losses are intrinsic physiological senescence and invasion by fungal pathogens. Anthracnose caused by *Colletotrichum s*pp is one of the common fungal diseases that can result in serious economic and extensive postharvest losses during transportation and storage of climacteric fruits (Bautista‐Baños et al., 2013; Pavitra Kumari & Singh, 2017). Symptoms of anthracnose postharvest are sunken black spots that occur on the fruit surface during ripening (Tian et al., 2016). Mango anthracnose, caused by *Colletotrichum gloeosporioides*, is a significant threat to farmers around the world, as it results in massive pre‐ and postharvest losses in mangoes (Lima et al., 2013; Pavitra Kumari & Singh, 2017). During postharvest preservation and following export, papaya deteriorates, primarily due to anthracnose caused by *Colletotrichum fructicola* and *Colletotrichum gloeosporioides* Penz (Madani et al., 2014; Vilaplana et al., 2020). The most damaging postharvest disease of bananas is anthracnose, which is caused by the fungus *Colletotrichum musae* leading to substantial economic loss (Khaliq et al., 2019). It degrades the fruit's quality and nutritional value and makes it unfit for marketing and consumption, resulting in significant losses for farmers and traders.

The use of synthetic chemical fungicides has resulted in issues with postharvest disease control, including decreased efficacy, increased plant pathogen resistance to active ingredients, environmental damage, and a serious negative impact on human health (Dubey et al., 2007; Kumar & Kudachikar, 2018). Synthetic chemicals have been used to reduce fungi attacks in postharvest storage of fruits, but there are concerns against their safety due to the toxicity of chemicals thus making it an urgent need to find alternative environmentally friendly technologies. With the above‐mentioned problems facing climacteric fruits, one of the most preferred solutions includes the application of edible coatings (Romanazzi et al., 2016). Many studies have shown that edible coatings made from a variety of biopolymers can effectively preserve the nutritional properties and extend the shelf life of climacteric fruits. To suppress decay, improve fruit quality, and prolong the shelf life of climacteric fruits during postharvest storage, edible coatings such as chitosan, aloe vera gel, and gum arabic are widely used (Berumen‐Varela et al., 2015; Khaliq et al., 2019; Maqbool et al., 2010).

## 3 FORMULATION OF EDIBLE COATINGS

Generally, films from edible coatings are prepared from polymers such as hydrocolloids (polysaccharides and protein), lipids, a combination of both (referred to as composite coatings), and the addition of plasticizers (Dhall, 2013). Edible coatings can be applied over the food product in liquid form by spraying, extrusion, solvent casting, brushing, or dipping to achieve a thin protective layer (Thakur et al., 2019; Yousuf et al., 2018). A review by Nor and Ding (2020) compiles all possible coatings that are functional for tropical fruit. The review also covers the fundamentals of coating attributes, materials, and processes, which include the following: the effectiveness of various coating materials such as polysaccharide, protein, lipid, and composite‐based coatings has been highlighted, and various application methods, and coating protection. Dhaka and Upadhyay (2018) wrote a simple review on edible films and coatings, as well as recent innovations in the field. This analysis provided a detailed overview of various aspects of edible films and coatings, as well as a discussion of current trends and innovations. Furthermore, edible coatings can be formulated using polymeric materials derived from both plant and animal sources. The application of animal extracts in the production of edible coatings has been limited due to allergies from animal foods and the waxy nature of coatings thereby limiting food product acceptability compared with plant extracts with medicinal benefits (Flores‐López et al., 2016; Oms‐oliu et al., 2010). Plant extract‐derived edible coatings can delay ripening, improve esthetic appearance by shinning the produce and covering minor scars (Murmu & Mishra, 2018). Also, they are an inexpensive means for maintaining the quality of fresh produce. The use of edible coatings from plant extracts has been proposed to decrease the usage of nonbiodegradable storage polyethylene plastic films and containers, thereby reducing pollution to the environment (Bourtoom, 2008).

Plant extracts with high antioxidant properties can also improve the nutritional qualities of fruits. The effect of plant edible coatings on the quality attributes and nutritional characteristics of various climacteric fruits such as banana, apple, mango, and papaya has been studied. A review by Ncama et al.  (2018) gave a comprehensive report on the use of plant extract‐derived edible coatings for both climacteric and nonclimacteric fruits. Some of the plants whose extracts are used as edible coatings for climacteric fruits include moringa leaf extract (Tesfay & Magwaza, 2017), corn starch and rice starch (Razak & Lazim, 2015; Thakur et al., 2019), aloe vera (Khaliq et al., 2019), and gum arabic (Maqbool et al., 2011). The use of natural edible coating extracts is one of the most promising technologies to enhance the protection and quality of fruits because it is considered as being environmentally friendly and acceptable for consumers (Janisiewicz & Korsten, 2002).

### 3.1 Unique features of edible coatings

The edible coating serves as a barrier to control moisture loss and gas exchange (CO_2_ and O_2_) between the fruit and their surrounding environment thereby slowing down the rate of respiration, retarding the physiological ripening process, and preventing the loss of natural volatile flavor compounds (Khatri et al., 2020; Pratiwi et al., 2015; Rojas‐Graü et al., 2009). Furthermore, edible coatings can safely be consumed as part of the product and contain health benefits because they are made of food‐grade products (Shit & Shah, 2014). Other advantages of coatings include their edibility and biodegradability, as well as the avoidance of waste and the commercialization of food without preservatives (Kumari et al., 2017; Tavassoli‐Kafrani et al., 2016). The coating enhances postharvest shelf life by delaying physicochemical changes and preventing the development of physiological disorders. Likewise, edible coatings have a high propensity to provide active compounds such as antioxidants (ascorbic acid, citric acid, and oxalic acid), antimicrobials (potassium sorbate, and essential oil), texture enhancers (calcium chloride, calcium lactate, and calcium gluconate), and nutrients (Vitamin E), which can improve resistance to fungal pathogens plus the dietary and organoleptic characteristics of fruits (Arnon‐Rips et al., 2019; Dhall, 2013; Martín‐Belloso et al., 2009). To choose a coating for fruits, it is important to understand the properties of the coatings and how well they interact with the fruit surface and surroundings during storage. Edible coatings need to have a flawless adhesion capacity, high microbial protection, moisture exchange properties, appealing esthetic appearance, and above all availability at an affordable price (Poverenov et al., 2014). It is advisable to use coatings that have been shown to remove respiration peaks efficiently and reduce the output of ethylene to a minimal level.

### 3.2 The effect of edible coatings on quality attributes in climacteric fruits

The ripening process of climacteric fruit shows a dramatic increase in ethylene production and respiration rate at the onset of ripening. Various parameters such as weight loss, firmness, total soluble solids, total phenols, and antioxidant activity, decay rate, and shelf life have been used to assess quality in climacteric fruits (Hudina et  al., 2012). The efficacy of using edible coatings has been demonstrated by increasing evidence from numerous studies. In a study that investigated the effect of shellac and gelatin composite coatings for extension of shelf life of a banana, Soradech et al.  (2017) observed 60% of shellac and 40% gelatin act as an effective physical barrier around the fruit, resulting in a slow decrease in weight loss, and softening. The quality was maintained for more than 30 days compared with uncoated banana. A report by Jaiswal et al.  (2018) indicated that the incorporation of citric acid and neem extract improved the effectiveness of aloe vera by maintaining the firmness, color, sensory attribute, and market value of tomato fruit. Aloe vera (40%) plus citric acid gave the best result compared with other concentrations (20%, 60%, and 80%). Recently, a study by Kubheka et al.  (2020) showed the effect of gum arabic‐ and Carboxymethylcellulose‐containing moringa leaf extract on maintaining quality and control of *C. gloeosporiodes* on maluma avocado at cold storage for 21 days. Based on the result, 15% gum arabic plus moringa followed by 10% gum arabic and moringa and 1% Carboxymethylcellulose plus moringa were the most effective in reducing weight and firmness loss. The coatings also delayed color change and inhibited the growth of *C. gloeosporiodes* respectively compared with the control.

Edible coatings containing antioxidants and antimicrobials have been shown to improve the nutrient value and protect against pathogens and spoilage (Arnon‐Rips et al., 2019; Dhall, 2013; Pranoto et al., 2005). A report by Yang et al.  (2019) evaluated the efficacy of gum arabic enriched with white and red roselle extract on the postharvest quality of blueberry fruit stored at refrigerator temperature. Gum arabic was found to have antioxidant and antimicrobial effects, owing to polyphenol compounds in the gum. The existence of more bioactive compounds, such as phenolic and flavonoid compounds, which could inhibit certain microorganisms, could explain the red roselle extract's higher antimicrobial capacity against all tested microorganisms. Due to the synergistic effects of natural antimicrobial compounds in gum arabic and roselle extracts, this finding showed the least decay. Gum arabic combined with roselle extract served as a barrier between the blueberries and their surroundings, controlling gas and water vapor exchange and delaying weight loss during storage. The slower loss of firmness of coated fruits may be linked to a well‐maintained cellular membrane as a result of gum arabic, gum arabic + white roselle extract, gum arabic + red roselle extract coatings inhibiting PPO enzyme activity, reducing the softening process of the fruit. Therefore, the addition of white and red roselle to gum arabic showed a better performance in maintaining firmness, reducing weight loss, and decay percentage compared with gum arabic alone and the control. Edible coating containing gum ghatti enriched with clove oil was used on banana and papaya fruits to extend shelf life. Gum ghatti (3%) plus clove oil (0.1%) retained the ascorbic acid, total phenols, and antioxidant activity in both fruits. In bananas, shelf life was extended by 3 days in both fruits relative to the control (Joshi et al., 2017, 2018).

A report by Abd El‐Razek et al.  (2019) showed that moringa and green tea extracts act as an antioxidant coating and was effective in reducing vitamin C loss, total soluble solids, total phenols, and antioxidant activity, and a decrease in weight loss was observed in mango fruits at 2, 4, and 6 weeks during two consecutive seasons. Moringa leaf extracts are also rich in antioxidants and have antibacterial effects against a range of microorganisms. The high phytochemical constituents of moringa plant extracts, which include phenols, alkaloids, and tannins among a few others, have been attributed to the inhibitory effect on the mycelial growth of various pathogens. Furthermore, tea leaves are high in polyphenolic compounds, which have a high antioxidant potential and antimicrobial activity in general; hence, the properties of moringa and green tea make them suitable as coating materials. Natural substances present in moringa and green tea extracts, which are high in antioxidants, serve as electron donors, creating free radicals that minimize normal respiration and transpiration, as well as enhance stomata closure. The reduction of fruit decay caused by the coating of moringa and green tea extracts is linked to a reduction in the activity of cell wall‐degrading enzymes, which prolongs the postharvest period and delays fruit ripening. Because of its low oxygen permeability, which decreased enzyme activity and prevented oxidation of vitamin C, moringa and green tea extracts as antioxidant coating treatments were successful in reducing vitamin C loss in mango fruits during all storage periods. The best result to achieve a high value of storability and quality was shown in applying 10% moringa leave extract followed by 5% green tea extract under refrigerated storage.

Shah and Hashmi (2020) investigated the impact of chitosan in combination with aloe vera gel on the storage life of mango fruits. They found that adding Chitosan to aloe vera lowered weight loss, respiration rate, and ethylene generation more effectively than using chitosan alone or control samples. Furthermore, the combination treatment preserved fruit quality metrics such as titratable acidity, total soluble solids, fruit firmness, ascorbic acid, and peel color. This study shows that combined application of chitosan and aloe vera synergistically improves the phenolic content of mango fruit, sustaining high ascorbic acid, total phenolic content, and antioxidant activity during storage. This suggests that addition of aloe vera may enhance the barrier of chitosan coating thereby improving antimicrobial properties and decreasing permeability to water and gaseous exchange (Mishra et al., 2010). Jodhani and Nataraj (2021) focus their research to study how aloe vera gel, lemon peel extract and their combination as edible coating treatment affect banana postharvest quality and shelf life when stored at room temperature. The consistency of bananas in treated fruits at the end of the storage period indicated that the lemon peel extract concentrate in the edible coating treatment prevents microbial contamination and protects the fruits from pathogenic fungi deterioration. When compared to the control, the coated banana has less weight loss and good firmness, as well as low infection, which is the most important factor that determines the banana's storage life and consistency. Aloe gel and LPE coating application dramatically reduced fruit respiration, slowed ripening and delayed the emergence of visual indicators of quality loss, all of which are undesirable to consumers. The aloe gel and LPE coating effectively reduced the rate of water loss from bananas during storage, according to these findings.

Daisy et al.  (2020) observed the effect of gum arabic on the shelf life and quality of mango fruit during storage at room temperature. Gum arabic coatings have been shown to have gas and water vapor barrier properties, allowing mango to last longer while preserving quality. Gum arabic changed the environment around the fruits by creating a semi‐permeable film that prevented moisture and gas loss through the coat thereby delaying ripening, and this allowed gum arabic‐coated mangoes to have a shelf life of 15 days compared to less than 10 days in control fruits. The efficacy of various plant edible coatings including gum arabic, sodium caseinate with the addition of lemongrass, and cinnamon essential oil was evaluated by Murmu and Mishra (2018) at varying concentrations on the postharvest quality attributes of guava fruit. The fruit with coating exhibited a slower rise in total and reducing sugars, lowest browning rate, higher retention of ascorbic acid, phenols and flavonoid content, overall sensory score, and extended shelf life by 33 days compared with the control. The addition of lemongrass oil and cinnamon oil helped retain higher membrane integrity thereby preventing disease occurrence and sustained metabolic rate.

Unfortunately, the application of edible coating still faces limitations: for example, they may be unattractive to the consumers as some exhibit their color or require undesirably high dose applications to be effective. Studies have shown nanomaterials with a unique character such as small size and quantum size have been explored to produce nanoparticles which can improve the efficacy of coatings (He & Hwang, 2016).

## 4 NANOTECHNOLOGY

Nanotechnology is another form of innovation that offers countless postharvest management approaches capable of producing new materials by reducing particle sizes to a nanometric scale (at least one‐dimensional ranges of 1–100 nanometers) giving materials with distinct and improved properties compared with larger ones (Magnuson et al., 2011; Parisi et al., 2015). Nanoparticles (NPs) (nanoscale structures with sizes ranging from 1 to 100 nm) have emerged as one of the outstanding nanotechnology discoveries designed to solve the day‐to‐day problems of the world today. Based on fundamental characteristics, nanoparticles exhibit an entirely new or enhanced properties, such as size, large ratio of surface area to volume distribution, and morphology. It is important to remember that only a small amount need to be added to form a strong interfacial contact with the edible coat polymer. The addition of nanoparticles to the edible coating results in a significant extension of fruit shelf life compared with the effect of the pure polymer without nanoparticles (Gad & Zag Zog, 2017). Nanoparticles, when added to edible coatings, can significantly improve the mechanical and barrier properties and increase thermal stability (De Moura et al., 2009; Shankar & Rhim, 2015). Nanoparticles can be classified into two groups namely organic NPs and inorganic NPs. More emphasis is placed on inorganic nanoparticles because of their stability compared with organic ones that are heat labile compounds. Inorganic nanoparticles consist of metal or metal oxides, such as gold (Au), silver (Ag), iron oxide (Fe3O4), titanium oxide (TiO2), copper oxide (CuO), zinc oxide (ZnO), aluminum oxides, cerium dioxide hydroxides, calcium carbonate and carbon‐based materials (Bouwmeester et al., 2014; He & Hwang, 2016). According to numerous reports, using nanotechnology to extend the shelf life of fruits is one of the most effective techniques (Bhusare & Kadam, 2020; Flores‐López et al., 2016; Ijaz et al., 2020; Lloret et al., 2012; Ruffo Roberto et al., 2019). The most used method for the application of nanoparticles is the dipping method (Table 1), by immersing the fruit into the coating solution forming a thin layer on the surface of fruits and subjecting it to cold or ambient temperature storage. A review by Rajat Suhag et al.  (2020) reporting on film formation and deposition methods of edible coating on food products suggested that the dipping method was found to be the cheapest and easiest to use on the surface of food products among other edible coating methods.

**TABLE 1:** Nanoparticle‐enriched edible coatings applied on popular climacteric fruits

Fruit type	Nanoparticle components	Other ingredients	Coating method	Effect	References
Apple	Chitosan	Acetic acid	Dipping	The coating, improved consistency of color quality, slowed down fruit softening and decreased weight loss by up to 2.5 times over 9 weeks of storage	Gardesh et al. (2016)
	Silver/Zinc oxide	Gelatin/Chitosan	Dipping	The fruit quality was preserved, and the shelf life was extended by 42 days	Bakhy et al. (2018)
Tomato	Gum Arabic	Tween & NaCI	Dipping	It maintained overall quality and extended the storage life by 14 days.	Paladugu, and Gunasekaran, (2017)
	Silver	Silver nanoparticles from Chinese tea	Dipping	Specifically found to reduce weight loss in fruit, and extended shelf life of fruit by 18days of storage at room temperature	Gao et al. (2017)
	Silver/Zinc	Gelatin/Chitosan	Dipping	The fruit quality was maintained, and the shelf life of coated fruit was extended by 63 days	Bakhy et al. (2018)
	Zinc	Carboxymethylcellulose	Dipping	The combination showed a beneficial effect in improving quality parameter compared with control and effectively delayed the disease severity during 15 days of storage	Saekow et al. (2019)
Banana	Zinc oxide	Soybean protein isolate & cinnamaldehyde	Dipping	It effectively delayed ripening and improve the shelf life of banana by maintaining the nutrient content and hinder the loss of water during 7 days of storage	Li et al. (2019)
	Chitosan	Chitosan	Dipping	The coatings maintained the sensory quality and extended the shelf life of banana for several days	Lustriane et al. (2018)
	Chitosan	Acetic acid	Spraying	The ripening was delayed by showing a slower rate of skin discoloration as compared to control during 6 days of storage	Esyanti et al. (2019)
	Zinc	Chitosan/gum Arabic	Dipping	The consistency of the bananas was retained for a slightly longer period, and shelf life was prolonged after more than 17 days in storage	La et al. (2021)
Fresh produce	Nanoparticle components	Other ingredients	Coating method	Effect	References
Banana	Silver	Neem and Ajwain	Spraying	Control Anthracnose disease in banana	Jagana et al. (2017)
Mangoes	Calcium	Ascorbic acid	Dipping	It alleviates internal browning and maintains the phenolic compound of mangoes during cold storage	Lo'ay et al. (2019)
	Zinc	Carrageenan	Dipping	Increase antimicrobial properties and maintain the shelf life of whole mango fruit	Meindrawan et al. (2018)
	Silver	Chitosan and Tween 80	Dipping	The combination minimized postharvest decay by inhibiting anthracnose incidence on mango during 7 days of storage	Chowdappa et al. (2014)
	Zinc	Aloe vera gel & glycerol	dipping	It improves quality parameter during 9 days of storage	Dubey et al. (2019)
	Zinc	Cassava starch & stearic acid	Dipping	It was effective in reducing weight loss, delayed microbial growth, and improving the shelf life of fresh‐cut mango during storage at 8°C, for 12 days.	Luliani et al. (2018)
Fig	Zinc	Chitosan & acetic acid	Dipping	Coating delayed the ripening of fruits and keep quality during storage	Lakshmi et al. (2018)
Guava	Chitosan	Xanthan gum & tween	Dipping	It enhances overall quality during cold storage and shelf life periods	Gad & Zag Zog, 2017
Papaya	Silver	hydroxypropyl methylcellulose	Dipping	Silver nanoparticle was effective against *Colletotrichum gloeosporioides,* preserved postharvest quality, and shelf life was extended by 14 days during storage	Vieira et al. (2020)
Apricot	Silver	Glycerol	Dipping	It significantly reduces weight loss, decay percentage, and kept the quality for 24 days at 6°C	Shahat et al. (2020)

### 4.1 Synthesis of nanoparticles

Currently, various physical and chemical methods are widely used to synthesize nanoparticles, enabling particles with the necessary characteristics to be obtained. These manufacturing methods can present several drawbacks such as the use of nonbiodegradable stabilizing agents, labor‐intensive, usage of toxic chemicals, and are potentially detrimental to the environment and living organisms (MubarakAli et al., 2015; Phanjom & Ahmed, 2015). Therefore, to minimize hazards to the environment, green/biochemical synthesis of nanoparticles offers an appealing means for nanoparticle synthesis and promises to help overcome these physical and chemical disadvantages (Nayak et al., 2015; Shankar et al., 2004). This is due to low synthesis costs, short development time, easy accessibility, eco‐friendliness, economic considerations (have the potential to generate high production volumes), and use of plant‐based materials (Akintelu & Folorunso, 2019; Kavitha et al., 2013). The green synthesis technique involves the use of naturally existing resources where an extract of plants acts as a reducing and stabilizing agent (Jamdagni et al., 2018; Sharma et al., 2016).

The green synthesis of different nanoparticles based on plant extracts has been extensively studied since the last decade. Many studies on the production of silver nanoparticles from plant extracts have been reported by (Ahmad et al., 2019; Rajeshkumar & Bharath, 2017; Srikar et al., 2016). A thorough examination of the green synthesis and characterization methods for ZnO NPs derived from various biological sources was studied and has thus become a major research subject (Agarwal et al., 2017; Ahmed et al., 2017; Bandeira et al., 2020). Alternatively, nanoparticles can be synthesized with the use of natural polymers from marine (chitin and chitosan) or agricultural waste (cellulose, gums, starch, and pectin) which has the added advantage owing to their stability, small size, edibility, and nontoxic nature (He & Hwang, 2016).

## 5 NANOPARTICLES COMMONLY USED IN CLIMACTERIC FRUIT

Several nanoparticles have been used in fruits. The most explored nanoparticles in climacteric fruits are zinc oxide, silver, and chitosan considering their higher antimicrobial activity and stability. Nevertheless, other nanoparticles such as iron, titanium dioxide, cerium oxide, and copper have been used in various field of the food sector. Titanium dioxide nanoparticle was reported used with chitosan coating to form a film on the surface of mango fruit. It was effective in reducing losses caused by decay, delay respiration, and maintain the firmness of fruits (Xing et al., 2020). Copper nanoparticles were sprayed on tomatoes and show an increase in the content of bioactive compound and maintenance of quality (López‐Vargas et al., 2018). Also, cerium oxide was blended with ascorbic acid exhibiting a significant reduction of internal browning of mango fruit during cold storage. In addition, Nanoparticles have been used in food packaging and additives but there are limited studies on their application on climacteric fruits; therefore, there is a need to explore the use in fruits shelf life extension (Kumar et al., 2020).

### 5.1 Chitosan nanoparticle

Chitosan (CS) is one of the promising biopolymers that has been studied as a nanoparticle because of its film‐forming capability, biodegradability, biocompatibility, and antimicrobial activity, nontoxic to humans, ease of alteration, and flexible physical and chemical properties (Divya & Jisha, 2018; Jianglian & Shaoying, 2013). Chitosan derived from the deacetylation of chitin in an alkaline medium is obtained from the waste products of the shellfish industry (Suseno et al., 2014; Xu et al., 2005). Chitosan is considered generally regarded as safe (GRAS) and recently approved by the United States Food and Drug Administration (USFDA) [Katiyar et al., 2014; Hu et al., 2019]. Chitosan nanoparticles (CS‐NPs) are a derivative of CS with excellent physicochemical properties (Divya et al., 2017; Kassem et al., 2019).

The chitosan nanoparticles outperformed the chitosan edible coatings in terms of antioxidant and antibacterial activity. CS‐NPs have a smaller particle size and a larger contact area than CS, which contributes to their high biological activity, and the CS‐NPs can move through biofilms and destroy pathogenic bacteria because of the same reasons (Huang & Li, 2014; Qi et al., 2004; Shrestha et al., 2010). The potential advantages of chitosan nanoparticles over conventional chitosan are distinct as they strengthen the barrier properties and functionality of edible coatings because of their increased surface area (Eshghi et al., 2014). Chitosan nanoparticles have the distinctive characteristics of chitosan biopolymer and are considered to have higher antimicrobial activities and barrier properties (Kalaivani et al., 2020). In previous research, a nanochitosan‐based coating was successfully applied on apples and bananas (Esyanti et al., 2019; Gardesh et al., 2016; Lustriane et al., 2018). This form of coating greatly enhanced sensory efficiency, increased storage life, and retained the fruit's bioactive components.

### 5.2 Zinc oxide nanoparticle (ZnONPs)

Zinc oxide nanoparticles have gained the attention of many researchers for their unusual peculiar optical and chemical behaviors among the metal oxide nanoparticles, which can be easily tuned by adjusting the morphology. Within the broad family of metal oxide nanoparticles, zinc oxide nanoparticles have been used in various advanced numerous cutting‐edge applications, such as electronics, communications, sensors, cosmetics, environmental protection, biology, and the medical industry, and their food safety (GRAS‐Generally Recognized as Safe) has been properly approved by the US Food and Drug Administration (FDA) (Noshirvani et al., 2017; Rasmussen et al., 2010). Due to its excellent mechanical properties, barrier capacities, biocompatibility, and antimicrobial broad‐spectrum performances, the zinc oxide nanoparticle (ZnONP) has gained considerable interest in sciences (Yusof & Zain, 2019). The antimicrobial properties of ZnO particles are due to the reactive oxygen species that form on their surface. In addition, recent scientific studies have shown that zinc is a promising coating material due to its being a relatively potent antimicrobial agent with high stability as a comparison to natural‐based coating, and there is no possible risk to human health from its use (Sun et al., 2018). And, as stated elsewhere, ZnONP's addition to polysaccharides, lipids, and protein‐based biopolymers can effectively improve the mechanical properties, barrier capacities, and physicochemical properties of edible coatings (Muraleedaran & Mujeeb, 2015; Wu et al., 2019). ZnONPs have highly effective antibacterial activity and are considered as a possible additive to replace hazardous chemicals and physical antibacterial materials (Awwad et al., 2020).

### 5.3 Silver nanoparticles (AgNPs)

Among nanoparticles, silver nanoparticles (AgNPs) are one of the most studied as they have been shown to be efficient against different microorganisms and are safe for humans (Aadil et al., 2018). Around the same time, silver has been adopted as an antimicrobial material that is relatively free of adverse effects. A wide variety of antibacterial, antifungal, and antiviral properties are found in silver nanoparticles. Due to its biocidal activity against a wide range of Gram‐positive and Gram‐negative microorganisms, yeast, molds, and viruses, silver is currently the most researched antibacterial nanoparticle. The release of Ag+ions, which bind to electron donor groups in molecules containing sulfur, oxygen, or nitrogen, is primarily responsible for the antimicrobial activity of silver nanoparticles. Additionally, AgNPs outperformed metallic silver in antimicrobial properties due to their incredibly large surface area, which allows for better contact with microorganisms (Toker et al., 2013). The safety limit of silver declared by EU safety regulations for foods is 0.05 mg/kg (Fernández et al., 2009). It is proved that a silver concentration of 0.06 mg/L is acceptable for coating fruits and vegetables (An et al., 2008). The use of silver nanoparticles, which include a wide variety of compounds that can be used in the formulation of edible coatings, is the most recent breakthrough advancement in the application and development of edible coatings for fresh fruit.

## 6 APPLICATION OF NANOPARTICLE‐ENHANCED EDIBLE COATINGS ON CLIMACTERIC FRUITS

The application of nanoparticle‐enhanced edible coatings has been explored in postharvest shelf life research and can be effective in improving color quality, firmness, increase antimicrobial properties, control enzymatic activity, and reducing weight loss of fruits (Table 1). The incorporation of silver nanoparticle into sodium alginate inhibits the growth of the microbial diseases in pear; because after coating, the silver nanoparticle‐incorporated sodium alginate coatings maintain its antibacterial activity against Gram‐positive and Gram‐negative bacteria. The coated fruit was found to be suitable for up to 10 days in storage as judged by the color and appearance, texture, and aftertaste compared with sodium alginate‐coated and ‐uncoated fruit (Mohammed Fayaz et al., 2009). Arroyo et al.  (2020) investigated the effect of chitosan plus alginate plus zinc nanoparticles on the postharvest life of guava. The coating was able to prevent rot appearance, retarded physiochemical changes related to maturation. Zinc nanoparticles combined with 90 or 100% chitosan, and 10% alginate extended the shelf life by 13 days compared with the control in guava.

The utilization of nanoparticles shows improvement in the storage quality of fruits. Joshy et al.  (2020) applied novel zinc oxide nanoparticle‐reinforced xanthan gum on apple and tomato fruit. It preserved the fruits from deterioration and water loss. Based on these findings, the manufactured edible coatings may serve as a novel antimicrobial agent to safeguard the fruit from microbial contamination. Malek (2020) observed the effect of storage temperatures on the shelf life of golden lily mangoes treated with zinc oxide nanoparticle and tapioca starch for 7 days of storage at 32°C, 27°C, and 5°C. The fruit firmness was reduced at 32°C, but storage life was 2 days maximum while storage temperature of 5°C was found to be most suitable for delaying textural changes and maintaining the storage life of mango by 7 days. The study's most striking finding is that thanks to zinc oxide's relatively strong antimicrobial properties and starch's good mechanical properties, the ZnO–starch coatings reduced anthracnose disease growth, delayed texture changes, and maintained the shelf life of mango at lower storage temperatures.

Chandirika et al.  (2018) studied the effect of the silver nanoparticles on the quality attributes of tomato fruit at room temperature. Their results indicated that the application of silver nanoparticles showed an extended shelf life period from 16 to 21 days and sensory quality was maintained when compared with control (noncoating). Li et al.  (2019) successfully developed zinc nanoparticles with soybean protein isolate and cinnamaldehyde as a coating for banana stored at room temperature. Results concluded that the coatings not only delayed ripening and extended shelf life up to 7 days in storage at room temperature but also inhibited fruit fungus spoilage through the oxidative stress‐directed manner. Table 1 shows more studies done on the application of nanoparticles to edible coating resulting in keeping the quality of climacteric fruits.

## 7 ADVANTAGES AND DISADVANTAGES OF ADDING NANOPARTICLES TO EDIBLE COATINGS

The benefits and drawbacks of the addition of nanoparticles to edible coatings have been studied. The addition of nanoparticles to edible coatings has provided various benefits, such as increased antimicrobial activity, and formation of stronger coating homogeneity on fruit surface (Acevedo‐Fani et al., 2017; Severino et al., 2014). Chitosan, zinc oxide, and silver are the most widely used nanoparticles in climacteric fruits that have shown promising effects when applied to edible polysaccharide and protein materials. It has been stated that zinc has better compatibility and heat resistance in climacteric fruits (Table 1). The main goal of adding nanoparticles to edible coatings is to enhance the biopolymer's mechanical properties and water vapor barrier.

Firstly, ZnO‐enhanced xanthan hybrid method provides greater health benefits considering zinc requirement in the human body and is healthy in blood compatibility and toxicity tests (Joshy et al., 2020). Zinc oxide metal has been shown to have antimicrobial properties, demonstrating strong effectiveness in inhibiting the growth of pathogenic microorganisms, even when added in small amounts such as 0.1%–0.5% (w/v) (Esparza‐González et al., 2016).

Recently, Meindrawan et al.  (2018) found that the addition of zinc nanoparticle to carrageenan effectively decreases weight loss and total acidity, preserve firmness, and delay discoloration and decay of mango fruit compared with carrageenan alone. This is because zinc can improve the gas barrier of the coating as compared to carrageenan alone which tends to be hydrophilic. Similarly, zinc significantly improves the quality of cherry tomatoes by suppressing their respiration and water evaporation thus ensuring a better preservative effect at room temperature storage. The addition of zinc to carboxymethylcellulose and cinnamaldehyde not only reduced weight loss and ensured fruit firmness for a longer period but significantly inhibited the tested fungi showing greater antimicrobial activity compared with noncoated or pure carboxymethylcellulose with cinnamaldehyde (Guo et al., 2020). It is possible to attribute this effect to the synergistic antifungal effect between ZnONPs and carboxymethylcellulose. Gad and Zag Zog (2017) tested xanthan gum mixed with 0.2% and 0.4% chitosan nanoparticles against the uncoated and xanthan alone on guava fruit. Xanthan gum mixed with 0.2% nanochitosan decreased decay, color change, maintained fruit firmness, vitamin C, and good taste compared with xanthan gum or a high concentration of chitosan nanoparticles (0.4%). It also enhances the overall quality and extended shelf life at cold storage. This recent utilization of nanoparticle has encouraged the use of a lower concentration of coating administered in form of nanoparticle to enhance quality and extend the shelf life of fruits.

Chitosan nanoparticles have demonstrated significant effects as a postharvest treatment in terms of antioxidant, antibacterial, and antifungal activities compared with chitosan (Avelelas et al., 2019; Divya et al., 2017). Compared with the use of chitosan, chitosan nanoparticles are more active and perform better, which is due to smaller particle size and increased nanoparticle contact area (Orellano et al., 2019; Qi et al., 2004). The size reduction of chitosan to a nanoscale can improve the functionality and properties at lower concentrations (Eshghi et al., 2014). The effective concentration of chitosan decreased significantly to 0.5% when used in nanoparticle form as compared to the higher amount suggested in previous studies for coating fruits (Esyanti et al., 2019). Chitosan alone or with other polymer has been used at a concentration as high as 2% on fruit to effectively preserve the quality of fruit, but with the introduction of nanoparticle, a lower amount is required to effectively preserve the quality of fruits (Khatri et al., 2020; Suseno et al., 2014).

As the penetration and absorption of chitosan increase dramatically in the form of nanoparticles, the effective amount of chitosan used for coating fruits can be substantially or greatly reduced (Zahid et al., 2012). Jagana et al.  (2017) studied the impact of nanosilver concentration with plant extract of neem on anthracnose diseases in banana fruit. The nanoparticle, applied using a spraying method, was able to control anthracnose disease in banana even at a low concentration of 0.2%. This was because nanoparticles were able to penetrate microbial cells effectively showing complete inhibition of spore germination of *Colletotrichum musae*. Edible coatings such as plant extract may possess strong odor and flavor and may have a strong negative effect on the sensory properties of fruits thereby limiting their application. Silver nanoparticles on the other hand do not adversely affect the sensory characteristics of fruits and are more acceptable to consumers (Chandirika et al., 2018).

Consequently, the higher concentration of nanoparticles can cause physiological damage thereby changing the internal atmosphere of fruits, and increased chlorophyll degradation by enhancing fruit ripening (Zambrano‐Zaragoza et al., 2013). Developing a nanoparticle can cost a lot of money; thereby, it is advisable to synthesis the material via a green method by using plant extracts, which have a lower cost. Edible coatings such as alginate display low viscosity fluid when used with zinc nanoparticles leading to an inability of gas exchange between fruit and the environment. Chitosan on the other hand has shown synergy relations, emulsifying, and crosslinking abilities when used with zinc (Arroyo et al., 2020). Therefore, some nanoparticles do not form a synergy effect with edible coatings, which might limit their application in postharvest treatment.

## 8 SAFETY CONCERNS AND LEGISLATION IN USING NANOPARTICLE‐ENHANCED EDIBLE COATINGS

Nanoparticles are used in the fruit industry for a variety of reasons, one of which is their unique properties, which are associated with their small size. Small particles, for example, are digested faster, have a greater surface reactivity, and can more efficiently penetrate biological barriers than larger particles. Currently, there is insufficient legislation regarding the use of nanoparticles in fruits, and consumers view emerging innovations as posing a danger to their health and the environment. Legislative barriers and uncertainty about the effectiveness of such systems, as well as their economic and environmental impact, may be the primary reasons for this. While legislation is still in its early stages, it must discuss all aspects of nanotechnology's use in the fruit industry around the world. Only the European Commission (EU) member states of Sweden, France, Denmark, Belgium, and Switzerland have adopted their regulations for nanomaterials or nano‐enabled goods at this time (Arts et al., 2015). Recent EU regulations mandated that any food ingredient derived from nanotechnological applications be subjected to a safety evaluation before being approved for use (Cubadda et al., 2013). Only a few nano‐form substances have made it to commercial applications thus far, particularly in the EU. The US Food and Drug Administration (FDA) has approved the use of many forms of nanoparticles, including Ag and titanium NPs, in commercially available products like antibacterial skin lotions and sunscreens. Nanoparticles have been used in the food industry for edible foods, providing some confidence that they can be used in fruits with a high degree of protection (Table 2). FresherLonger^TM^, BagsFresherLonger^TM^, Anson Nano Silver Fresh Containers, Nano Silver Food Container, Fresh Box Nano Silver Food Container, Miracle Food Storage, and Anson Nano Freshness‐Keeping Film are silver nanoparticles commercially available for use in countries such as the United States, China, and South Korea, and they are used in packaging of food products. With the ever‐increasing usage of nanoparticles on fruits on a commercial scale, it is critical to stress the value of performing short‐ and long‐term toxicity studies, both for the environment and for humans, to ensure consumer protection. Multiple factors influence the toxicity of NPs, including form, scale, surface charge, composition, and NP stability. The key danger is that nanoparticles used directly on fruit could migrate into the fruit product. More research demonstrating that the nano material does not migrate into the food matrix may help with regulatory and consumer acceptance. Also, the consequences of long‐term ingestion of low yet regular concentrations or doses of nanoparticles on fruits, on the other hand, have yet to be investigated. Risk assessments, biosafety, and legislation for inorganic nanoparticles are still a work in progress that necessitates further study. This reality motivates researchers to continue their research and development efforts in the field of nanotechnology in order to secure a more accurate understanding of the materials' applications, risks, and benefits in fruits during the postharvest stage.

**TABLE 2:** Application of nanoparticles in the food industry

Nanoparticles	Application in food industry	References
Zinc oxide	Active packaging for fresh orange juice	Emamifar et al. (2011)
Zinc oxide	Food additive	Pérez et al. (2011)
Zinc oxide	Antimicrobial food packaging	Suo et al. (2017); Beak et al., 2017
Zinc oxide	Antimicrobial agent	(Kim et al., 2020; McClements & Xiao, 2017)
Zinc oxide	Food packaging material	Espitia et al. (2016)
Zinc oxide	Food lining in packaging	Silvestre et al. (2011)
Silver	Antimicrobial agent in food packaging	Medina‐Reyes et al. (2020)
Silver	Surface coatings for sweets	Medina‐Reyes et al. (2020)
Silver	Antimicrobials in marine shrimp farming	Camacho‐Jiménez et al. (2020)
Silver Zeolite	Food preservation	Kawahara et al. (2000)
Silver	Antimicrobial packaging	Chaudhry et al. (2008)
Silver	Commercial food containers	Artiaga et al. (2015)
Silver	Commercial containers and bags	Ozaki et al. (2016)
Silver	Food storage and food packaging materials	
T_i_O_2_	Food colorant	Chen et al. (2017)
T_i_O_2_	Food additives	(EFSA ‐ European Food Safety Authority, 2000; Weir et al., 2012)
Zinc oxide and silver	Nanocomposite packaging for chicken	Panea et al. (2014)
Chitosan	Cheese, meat, and fermented sausage production	Wang et al. (2004)
Chitosan	Glazing material for frozen shrimp	Solval et al. (2014)
Gold	Food additives	(EFSA Panel (EFSA Panel o on Food Additives and Nutrient Sources added to Food), 2016)
Silicon dioxide	Anticaking and antifoaming agent in foodstuffs	(JECFA (Joint FAO/WHO Expert Committee on Food Additives), 2016)

Future research should focus on potential human health consequences, as certain materials, such as TiO2, have been linked to colon cancer. Deng et al. (2021) explored recent developments in the production of food nanoparticles as well as the possible threats and found that they pose a possible threat to the human gastrointestinal tract. To effectively avoid the potential risks of nanoparticle applications in the food industry, it is important to include a specific description that encompasses the unique properties of nanocomponents as well as the required application or limitations of nanomaterials for the related products. Finally, before nanomaterials can be commercialized, definitive and conclusive studies on their safety and environmental impact are needed (de Azeredo et al., 2018).

## 9 CONCLUSION AND FUTURE TRENDS

Climacteric fruits are a central component of the human diet, supplying important minerals and vitamins for human health. Efforts have been made to improve storage conditions of the fruits, monitor their susceptibility to disease after harvest, and preserve their freshness to meet consumers' demands. The acceptability of fruits by consumers depends on quality parameters such as color, texture, absence of decay, and most importantly, the nutritional and health benefit they provide. Edible coatings, driven by their low cost and nontoxic nature, are among the well‐studied natural polymers and their application has proven to be promising for fruit preservation. The application of nanoparticles appears to be highly promising in the field of postharvest storage for extending the shelf life of climacteric fruits. The current materials widely used for coating climacteric fruits are zinc oxide, silver, and chitosan nanoparticles because they show promising results in preserving the postharvest quality of fruits. This review has summarized that nanoparticle‐enhanced edible coatings applied to climacteric fruits can effectively improve their physical and sensory properties, inhibit the growth of microbes, and prolong the shelf life of fruits. When used singly, some edible coatings have shown unsatisfactory results in practical application; hence, their combination with nanoparticles helps to improve their physicochemical and biological properties.

There is a great potential to extend the use of other nanoparticles such as copper, cerium oxide, and titanium oxide as coating materials as they are of low concern. Also, food‐grade nanomaterials such as starch, cellulose, and gums are edible and nontoxic and hence present promising prospects for use in fruit coating. The combination of nanoparticle‐enriched edible coating with the use of existing technologies such as low‐temperature storage and controlled atmosphere storage is another great field of research. To understand the method of applying nanoparticle‐enhanced edible coatings and their effect on sensory and nutritional properties of climacteric fruits, further research is crucial. Nonetheless, despite research emphasis on improving the appearance of fruit, there is still a lot to be done towards improving the organoleptic properties and nutritive values through the reasonable application of food‐grade materials to synthesize nanoparticles. Again, further investigation is necessary regarding the behavior of these materials after ingestion and maximum allowable amounts of the nanoparticles that may be present in fruits to create a healthy nanoparticle that could be used on commercial products.

## CONFLICT OF INTEREST

The authors declare that they do not have any conflict of interest.

## ETHICAL APPROVAL

This study does not involve any human or animal testing.
